# Determination of Protein Interactions among Replication Components of Apple Necrotic Mosaic Virus

**DOI:** 10.3390/v12040474

**Published:** 2020-04-22

**Authors:** Zhen-Lu Zhang, Fu-Jun Zhang, Peng-Fei Zheng, Yin-Huan Xie, Chun-Xiang You, Yu-Jin Hao

**Affiliations:** State Key Laboratory of Crop Biology, College of Horticulture Science and Engineering, Shandong Agricultural University, Tai’an 271000, China; zhangzhenluwenchao@163.com (Z.-L.Z.); 17863805279@163.com (F.-J.Z.); zhengpengfei96@163.com (P.-F.Z.); 15505483595@163.com (Y.-H.X.); youchunxiang@sdau.edu.cn (C.-X.Y.)

**Keywords:** apple mosaic disease, apple necrotic mosaic virus, virus characterization, protein interaction, protein localization

## Abstract

Apple mosaic disease is one of the most widely distributed and destructive diseases in apple cultivation worldwide, especially in China, whose apple yields account for more than 50% of the global total. Apple necrotic mosaic virus (ApNMV) is a newly identified ilarvirus that is closely associated with apple mosaic disease in China; however, basic viral protein interactions that play key roles in virus replication and the viral life cycle have not been determined in ApNMV. Here, we first identify an ApNMV–Lw isolate that belongs to subgroup 3 in the genus *Ilarvirus*. ApNMV–Lw was used to investigate interactions among viral components. ApNMV 1a and 2a^pol^, encoded by RNA1 and RNA2, respectively, were co-localized in plant cell cytoplasm. ApNMV 1a interacted with itself at both the inter- and intramolecular levels, and its N-terminal portion played a key role in these interactions. 1a also interacted with 2a^pol^, and 1a’s C-terminal, together with 2a^pol^’s N-terminal, was required for this interaction. Moreover, the first 115 amino acids of 2a^pol^ were sufficient for permitting the 1a–2a^pol^ interaction. This study provides insight into the protein interactions among viral replication components of ApNMV, facilitating future investigations on its pathogenicity, as well as the development of strategies to control the virus and disease.

## 1. Introduction

Apple (*Malus domestica*) is one of the most important economic fruit trees that grow in many countries around the world, including China. However, fungi, bacteria, viruses, and viroids pose severe threats to the long-term survival of perennial woody plants such as apple trees. Important viruses and viroids of apple trees include apple chlorotic leaf spot virus (ACLSV), apple stem grooving virus (ASGV), apple stem pitting virus (ASPV), and apple scar skin viroid (ASSVd). Virus-induced diseases are one of the most important factors limiting apple production in China [1].

Apple mosaic disease was first reported in Europe in the 1930s and was transmittable by grafting [2]. The typical symptoms of apple mosaic disease only occur on leaves and not on the branches or fruits. The infected leaves usually display pale-yellow irregular spots or chlorosis, amorphous chlorotic bands along or between the veins, and mosaic patterns [3,4,5]. Currently, virus-induced apple mosaic disease is emerging as a severe threat to apple cultivation and production in many countries. For example, this disease caused reductions in apple yields by 30%–50% and 46% in India [6] and the USA [7], respectively. Photosynthetic deficiencies are the most likely mechanism underlying the yield reductions caused by apple mosaic disease, as previous work has shown that the net photosynthetic rate of leaves with mosaic symptoms is decreased to from approximately 3% to more than 45% [8].

In a long period, apple mosaic virus (ApMV), belonging to the genus *Ilarvirus* in the family *Bromoviridae* [9], was traditionally believed to be the sole causal agent of apple mosaic disease until prunus necrotic ringspot virus (PNRSV) [10,11], cucumber mosaic virus (CMV) [12], and apple necrotic mosaic virus (ApNMV) [13,14] were also reported to be associated with this disease. Specifically, 90% of the detected apple tree leaves (111 samples collected from different regions of China) with mosaic symptoms were infected with PNRSV, while ApMV was detected in none of them [11]. Furthermore, ApNMV was detected in 92.1% (268 out of 291 apple trees from different regions of China) of symptomatic trees, while ApMV was detected in none of them [14]. Moreover, the distribution of ApNMV was correlated with the symptomatic leaves, and these leaves were unevenly distributed in diseased apple trees [13]. In addition, ApNMV has also been reported to be isolated from crabapple (*Malus* spp.) leaves with mosaic symptoms [15]. Thus, in China, ApMV is not the only agent that can induce mosaic symptoms, as PNRSV and especially ApNMV, are also associated with apple mosaic disease. However, the induction of mosaic symptoms by ApNMV has not been verified via Koch’s Postulates [16], classical criteria for directly linking a specific pathogen with its corresponding symptom.

ApNMV was first reported to be associated with the apple mosaic disease by a Japanese group [13]. ApNMV, along with ApMV and PNRSV, is classified into subgroup 3 in the genus *Ilarvirus* in the family *Bromoviridae* [13]. Similar to other ilarviruses, ApNMV contains three positive single-strand genomic RNA segments (RNA1, RNA2, and RNA3) and a subgenomic RNA4 that derived from RNA3 [13,14]. The protein encoded by RNA1 contains an N-terminal methyltransferase (MET) domain and a C-terminal NTP-binding helicase (HEL) domain. RNA2 encodes the viral RNA-dependent RNA polymerase (RdRp), and both of the proteins encoded by RNA1 and RNA2 are responsible for viral replication [13]. RNA3 encodes the movement protein (MP), while the coat protein (CP) is encoded by a subgenomic RNA4. MP and CP are involved in the viral movement, including cell-to-cell movement and systematic transportation [13].

Here, we obtained the full genome sequences of ApNMV–Lw isolated from an orchard in Laiwu, China. The replication proteins of ApNMV are co-localized in the cytoplasm in plant epidermal cells. Meanwhile, ApNMV 1a interacts with itself, and the N-terminal MET domain plays a key role in 1a’s inter- and intra-molecular interactions. In addition, 1a’s C-terminal HEL portion interacts with the N-terminal of 2a^pol^. The first 115 amino acids (aa) of 2a^pol^ is sufficient for supporting the 1a–2a^pol^ interaction.

## 2. Materials and Methods

### 2.1. Plant Materials and Growth Conditions

Leaves with mosaic symptoms were collected from Fuji apple trees in an orchard in Laiwu, Shandong Province, China. Wild-type *N. benthamiana* were maintained in a greenhouse with a 16:8 h light:dark at 23 °C

### 2.2. Plasmid Construction and Genetic Transformation

All PCR products were first cloned into pEASY-Blunt Simple cloning vector (Transgen), and then were constructed to expression vectors. The 1a–GFP and 2apol–GFP constructs were generated using the modified pRI-101 vector [17]. cLuc–1a, 1a–nLuc, cLuc–2a^pol^, and 2a^pol^–nLuc were obtained by inserting 1a and 2a^pol^ into pGreenII 62–SK–nLuc and –cLuc, respectively [18]. For the BiFC assay, 1a was inserted into 35S::SPYCE–cYFP or 35S::SPYNE–nYFP to construct 1a–cYFP and 1a–nYFP, respectively [19]. 1a and 2a^pol^ were inserted into pGEX–4T–1 and pET–32a to obtain the GST- and HIS-tagged fusion proteins, respectively, for the pull-down assay. pGAD424 and pGBT9 were used to make the constructs in the Y2H experiments. The primers used to amplify the fragments and make constructs are shown in Table S1. Agrobacterium strain LBA4404 was used for agro-infiltration to achieve the transformation.

### 2.3. RNA Extraction and Complete Genomic RNA Sequence Acquisition

Total RNAs were extracted using the hot phenol method [20]. Briefly, the ground samples were treated with hot phenol and extracted with phenol/chloroform solution. Total RNAs were then precipitated by sodium acetate and washed with 70% ethanol. Lastly, RNAs were resuspended in RNase-free water.

First-strand cDNAs were synthesized with the PrimeScript RT reagent Kit (TaKaRa BIO INC, Otsu, Shiga, Japan) following the manufacturer’s instructions and using Random 6 as a primer. The middle fragments of RNA1, RNA2, and RNA3 were amplified by RNA1–F/R, RNA2–F/R, and RNA3–F/R primer pairs, respectively. The flanked 5′ and 3′ ends of each genomic RNA segment were amplified using the SMARTer RACE 5′/3′ Kit (Clontech Laboratories Inc, Mountain view, CA, USA) following the user manual. Finally, the three amplified fragments (5′ end, middle fragment, and 3′ end) were aligned and assembled to obtain the full length of each RNA segment, and, in turn, the full genome sequences of the ApNMV–Lw isolate. All of the primers used in genome RNA amplification are shown in Supplemental Table S2.

### 2.4. Homology Comparison and Phylogenetic Analysis

The similarity of ApNMV–Lw with other ilarviruses at the nucleotides levels of RNA1, RNA2, and RNA3 as well as the amino acid levels of 1a, 2a^pol^, MP, and CP were analyzed by DNAman (Lynnon Biosoft, San Ramon, CA, USA) software. Phylogenetic trees were constructed with MEGA (version 7.0.14, developed by Sudhir Kumar-the prject leader-from the Arizona State University, USA) software by using ClustalW and the neighbor-joining method [21] with 1000 bootstrap replications based on the nucleotide sequences of RNA1, RNA2, and RNA3 of ApNMV–Lw and other ilarviruses.

### 2.5. Yeast-Two-Hybrid Assay

The Y2H assay was conducted as previously described [22]. Briefly, the cDNAs of the target genes were amplified and inserted into the pGAD424 and pGBT9 vectors. The bait and prey constructs were transformed into the Y2H Gold yeast strain and grown on the selection medium without lucine (Leu) and tryptophan (Trp). The yeast colonies were then grown on SD base-Leu/-Trp medium and were transferred to selection medium without Leu, Trp, histidine, and adenine to characterize the interactions between bait and prey proteins.

### 2.6. Pull-Down Analysis

The pull-down assay was performed using the Pierce GST Spin Purification Kit per manufacturer’s protocol. The full-length cDNAs of ApNMV 1a and 2a^pol^ were amplified and inserted into pET–32a and pGEX–4T–1. The constructs were then transformed into *Escherichia coli* BL21 (DE3, Transgene) and treated with isopropyl β-D-1-thiogalactopyranoside for 4 h at 37 °C to induce the production of GST– and HIS–tagged fusion proteins, which were later used for the pull-down assay. The GST–1a recombinant protein was mixed with -HIS tagged proteins and then eluted with glutathione-agarose. The resultant protein mix was incubated at 4 °C for 1 h with gentle shaking and was then washed three times to remove the non-specific binding proteins. Finally, the agarose was eluted with solutions containing glutathione and boiled for 5 min. The supernatant was then used for sodium dodecyl sulfate-polyacrylamide gel electrophoresis and immunoblotted with antibodies.

### 2.7. Bimolecular Fluorescent Complementation (BiFC) Assay

The coding sequence of ApNMV 1a was constructed into 35S::SPYCE–cYFP and 35S::SPYNE–nYFP vectors [19]. The recombinant plasmids were transformed into Agrobacterium LBA4404 and co-infiltrated into *N. benthamiana* leaves. Two days post-infiltration, the YFP signals were observed under a confocal microscope (Zeiss, LSM880) and the “best signal” mode was selected to obtain different signals. eGFP, RFP, and DAPI signals were observed under excitations of 488, 594, and 405 nm, respectively. The images were acquired through single optical sections in the “Frame” Mode, with a scan speed of 6 (the scan time was about 10 s).

### 2.8. Luciferase Complementation Imaging Assay

The coding sequences of ApNMV 1a and 2a^pol^ were inserted into pGreenII 62–SK–nLuc and –cLuc vectors [18], respectively. The constructs were transformed into Agrobacterium LBA4404 and co-infiltrated into *N. benthamiana* leaves. Two days post-infiltration, the substrate of luciferase was sprayed on the leaves and maintained for 3 min. The samples were then observed under an in vivo imaging system (IVIS, Lumina II) that could detect bioluminescent signals. In the IVIS acquisition control panel, “Luminescent” was selected as the “Imaging Mode”, the exposure time was set to 30 s, and the photograph was set to “Auto”. After taking the photograph, the system allows the color scale to be adjusted (the minimum and maximum of the color scale), which is shown as a color bar on the right side of the image, so that the contrast of the image can be enhanced.

## 3. Results

### 3.1. Complete Nucleotide Sequence and Phylogenetic Analysis of ApNMV–Lw

**Figure 1:**
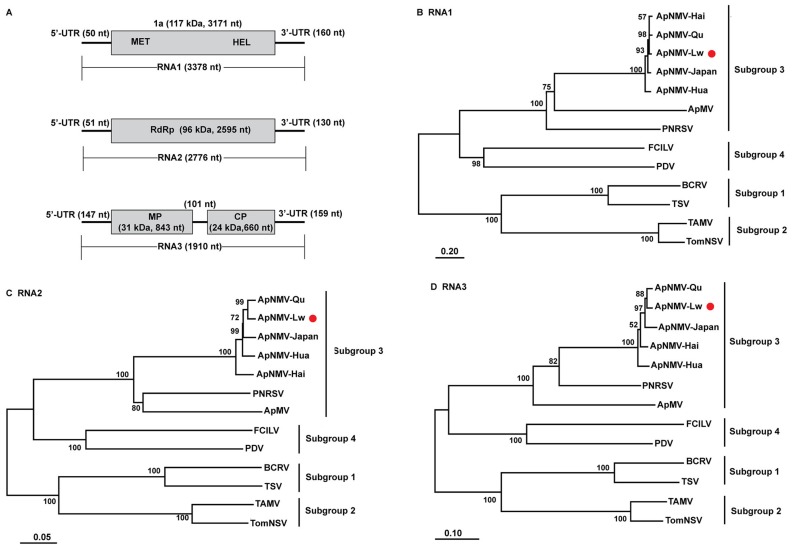
The genome organization of apple necrotic mosaic virus (ApNMV)–Laiwu isolate and its phylogenetic relationships with other members of *Ilarvirus*. (**A**) Genomic structure of ApNMV–Laiwu isolate. UTR, untranslated region; MET, methyltransferase; HEL, NTP-binding helicase; RdRp: RNA-dependent RNA polymerase; MP, movement protein; CP, coat protein. The phylogenetic trees of **B**–**D** were constructed using the neighbor-joining method based on the nucleotide sequences of RNA1, RNA2, and RNA3 of the ApNMV–Laiwu isolate (labeled by red spot) and other *Ilarviruses*, respectively. The blackberry chlorotic ringspot virus (BCRV, NC011553, NC011554, NC011555), and tobacco streak virus (TSV, KR017708, KR017709, and KR017710) represent Subgroup 1; the tulare apple mosaic virus (TAMV, NC00383, NC00384, and NC00385) and tomato necrotic streak virus (TomNSV, NC039074, NC039075, and NC039076) represent Subgroup 2; the apple necrotic mosaic virus–Qu isolate (ApNMV–Qu, MG924896, MG924899, and MG924902), ApNMV–Hua (MG924895, MG924898, and MG924901), ApNMV–Hai (MG924894, MG924897, and MG924900), ApNMV–Japan ( NC040469, NC040470, and NC040471), ApNMV–Laiwu (MN023210, MN023211, and MN023212), prunus necrotic ringspot virus (PNRSV, JN416774, JN416775, and JN416776), and apple mosaic virus (ApMV, NC003464, NC003465, and NC003480) represent Subgroup 3; the fragaria chiloensis latent virus (FCILV, NC006566, NC006567, and NC006568) and prune dwarf virus (PDV, NC008037, NC008038, and NC008039) represent Subgroup 4.

The entire genome sequence of the ApNMV–Laiwu isolate (ApNMV–Lw) was obtained by reverse transcription (RT)–PCR and the 3′-/5′-rapid amplification of cDNA ends (RACE) method. The genome contains three single positive-strand RNA molecules: RNA1, RNA2, and RNA3 (Figure 1A). RNA1 contains one open reading frame (ORF), starting from 51 nt and ending at 3221 nt, and is flanked by a 50 nt-long 5′-untranslated region (UTR) and 160 nt-long 3′-UTR. The protein (1a) encoded by RNA1 is 1056 aa in length, and the molecular mass is 117 kDa. The 1a protein has a conserved N-terminal MET domain and C-terminal HEL domain (Figure 1A). RNA2 has a 51 nt-long 5′-UTR, a middle ORF that starts from 52 nt and ends at 2646 nt, followed by a 130 nt-long 3′-UTR. RNA2 encodes the 2a polymerase (2a^pol^) and is characterized by the activity of the RdRp, which is 864 aa in length and has a molecular mass of 96 kDa. According to the well-studied model virus brome mosaic virus (BMV), a type member in the family *Bromoviridae* that has a similar genomic structure with ApNMV [23], the proteins encoded by ApNMV RNA1 and RNA2 likely play essential roles in viral genomic RNA replication. RNA3 contains two ORFs, the first one starts from 148 nt and ends at 990 nt, while the second ORF runs from 1092 nt to 1751 nt. Aside from the 147 nt-long 5′-UTR and the 159 nt-long 3′-UTR, RNA3 also has a 101 nt non-coding intergenic region (IR) located between the two ORFs. This non-coding IR of PNRSV has been reported to play a key role in initiating the transcription of RNA4 from RNA3 to generate the second ORF by viral replicase [24]. However, the length of the IR in our study is 101 nt, which is similar to the six isolates from China (98 to 103 nt) reported by Xing et al. [14], but different from that of a Japanese isolate (145 nt) [13]. The first ORF of RNA3 encodes a 31 kDa MP containing 280 aa, while the second ORF encodes a 24-kDa CP containing 219 aa. Based on previous studies of BMV, both MP and CP are thought to be involved in viral movement [23].

To determine the taxonomic position of ApNMV–Lw, as well as its phylogenetic relationships with ApMV and PNRSV (the other two viruses related to apple mosaic disease), the phylogenetic tree of *ilarviruses* was constructed based on nucleotide sequences using the neighbor-joining method (Figure 1B–D). ApNMV–Lw, along with other reported ApNMV isolates, ApMV, and PNRSV, all belonged to Subgroup 3. The phylogenetic trees indicated that ApNMV RNA1s were most closely related to ApMV, while RNA2s and RNA3s were most closely related to PNRSV.

To further investigate the relationships between APNMV–Lw, ApMV, and PNRSV, sequence alignments at both nucleotide and amino acid levels were performed among the three viruses (Table 1). RNA1, RNA2, and RNA3 of ApNMV–Lw were 59.44%, 58.02%, and 49.73% identical to these same sequences in ApMV and 64.00%, 58.20%, and 59.39% identical to those of PNRSV at the nucleotide level, respectively. In addition, the similarity of ApNMV–Lw’s 1a, RdRp, MP, and CP ranged from 40.54%–63.72%, and 55.83%–69.60% to those proteins of ApMV and PNRSV at the amino acid level, respectively. Thus, ApNMV–Lw is more closely related to PNRSV when compared with ApMV, which is inconsistent with the result of phylogenetic trees. We also aligned the sequences of ApNMV–Lw to those of ApNMV isolates from apple trees in China [15] and Japan [13] as well as from crabapple trees in China [15]. Similarities ranged from 88.47% to 97.37% at the nucleotide level, and 92.86%–99.53% at the amino acid level (Table 1), indicating that the genome sequence of ApNMV is highly conserved among different regions and hosts.

### 3.2. ApNMV 1a Colocalizes with 2a^pol^ in the Cytoplasm

**Figure 2:**
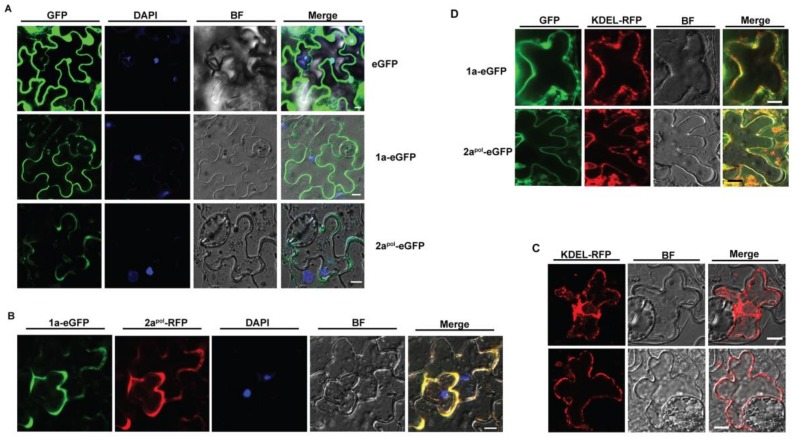
ApNMV 1a and 2a^pol^ co-localize in the cytoplasm. Confocal microscope images showing the localization (**A**) and colocalization (**B**) of 1a and 2a^pol^ in cells of *N. benthamiana* leaves. Unfused eGFP served as a control. (**C**) Distribution of Lys-Asp-Glu-Leu linked red fluorescent protein (KDEL–RFP) in the epidermal cells of *N. benthamiana*. (**D**) 1a–eGFP and 2a^pol^–eGFP partially co-localized with KDEL–RFP. Nuclei were stained with DAPI. BF, bright field; Scale bar, 10 μm. All the images in Figure 2 were obtained in a single optical section.

To visualize the intracellular distribution of ApNMV replication components, an enhanced green fluorescent protein (eGFP) was fused to both 1a and 2a^pol^ to obtain 1a–eGFP and 2a^pol^–eGFP recombinant constructs, respectively. The constructs were then transformed into *Agrobacterium rhizogenes* and infiltrated into *Nicotiana benthamiana* leaves. The signals were captured by a confocal microscope 2 days-post-infiltration (DPI). The eGFP protein was localized in both the cytoplasm and the 4′,6-diamidino-2-phenylindole (DAPI)-stained nucleus (top panel of Figure 2A). However, 1a-eGFP was only distributed in the cytoplasm (middle panel of Figure 2A), and 2a^pol^–eGFP showed a highly similar localization pattern with that of 1a–eGFP (bottom panel of Figure 2A).

Given that ApNMV 1a and 2a^pol^ are viral replication proteins and both of them are localized to the cytoplasm, we next asked whether they were co-localized in the cytoplasm. We fused red fluorescent protein (RFP) to 2a^pol^ and used the RFP signals as an indicator of the distribution of 2a^pol^. When 2a^pol^–RFP was expressed alone in *N. benthamiana* leaves, it localized in the cytoplasm, similar to 2a^pol^–eGFP (Figure S1). We then co-expressed the 1a–eGFP and 2a^pol^–RFP in *N. benthamiana* leaves using the agroinfiltration method and found that 1a–eGFP (the green signals) co-localized with 2a^pol^–RFP (the red signals) in the cytoplasm (Figure 2B).

Membrane-associated replication is one of the key common features of positive-stranded RNA viruses; thus, we next verified if ApNMV 1a, as well as 2a^pol^, was associated with endoplasmic reticulum (ER) membrane. We utilized a Lys-Asp-Glu-Leu (KDEL) residue-linked red fluorescent protein (RFP) as the ER marker (KDEL–RFP) (Figure 2C), and co-expressed 1a–eGFP and 2a^pol^–eGFP with KDEL–RFP, respectively. Both 1a–eGFP (upper panel) and 2a^pol^–eGFP (bottom panel) were partially overlapped with the KEDL–RFP (Figure 2D), suggesting they partially localized in cellular ER membranes.

### 3.3. Inter-Molecular Interactions of ApNMV 1a with Itself

Brome mosaic virus (BMV), a well-studied positive-strand RNA virus, is a typic member of the family *Bromoviridae*. Its genomic structure is similar to ApNMV. BMV 1a is a multifunctional protein, and its inter- and intramolecular interactions with itself play critical roles in the assembly of the viral replication complex (VRC) and viral genome replication [25]. Given that ApNMV belongs to the same family as BMV, and that the ApNMV 1a protein is also characterized by having an N-terminal MET domain and C-terminal HEL domain that is very similar to those of BMV 1a (Figure S2), we hypothesized that ApNMV 1a may also interact with itself and the RNA2-encoded protein.

**Figure 3:**
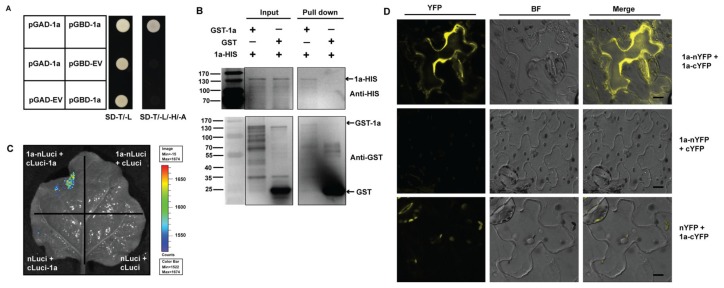
Self-intermolecular interactions of ApNMV 1a. **(A**) The yeast-two-hybrid assay indicated the presence of 1a–1a interactions. pGAD and pGBD empty vectors (EV) served as controls. (**B**) A pull-down assay also suggested the presence of 1a–1a interactions. The recombinant GST–1a and 1a–HIS proteins were mixed and incubated. The anti-GST and anti-HIS antibodies were used to detect the target proteins from both inputs and were pulled down. Molecular mass markers are shown and labeled (KDa) on the left side. GST–1a, GST, and 1a–HIS bands are indicated by arrows on the right side. (**C**) A luciferase complementation imaging assay verified 1a–1a interactions. The bar on the right side represents the intensity of the signals captured. The empty nLuci and cLuci vectors served as controls. (**D**) The bimolecular fluorescence complementation assay suggests that the 1a–1a interactions occurred in the cytoplasm of epidermal cells of *N. benthamiana* leaves. nYFP and cYFP empty vectors served as controls. BF, bright field; Scale bar, 10 μm. All images in Figure 3D were obtained in a single optical section.

To investigate the intermolecular interactions of ApNMV 1a with itself, we first utilized the yeast-two-hybrid (Y2H) method to perform an experiment. We constructed 1a to pGAD- and pGBD- vectors, and used empty vectors as controls. The yeast grew on SD–T/–L/–H/–A medium only when 1a was present in both vectors, indicating the presence of self, intermolecular interactions among 1a (Figure 3A). To further confirm the 1a–1a interactions, we next performed the in vitro pull-down assay. We first obtained the GST- and HIS-tagged 1a fusion proteins with a prokaryotic system. Next, the GST–1a was incubated in vitro with 1a–HIS protein, and the products were eluted with a glutathione column and immunoblotted with anti-GST and anti-HIS antibody. Empty GST served as the negative control. After traveling through the glutathione column, 1a–HIS was pulled down in the presence of GST–1a, but not in the GST control, indicating the presence of self, physical intermolecular interactions of 1a in vitro (Figure 3B).

Next, we used the luciferase complementation imaging assay to test for the presence of 1a–1a interactions in vivo. We first assembled 1a to the split of the N- and C- terminal of firefly luciferase protein to generate 1a–nLuc and cLuc–1a constructs, and co-injected the two constructs into *N. benthamiana* leaves via agroinfiltration method. The substrate of luciferase was sprayed on the leaves 2 DPI, and the samples were observed under an in vivo imaging system. Strong bioluminescence signals could only be captured only when both 1a–nLuc and cLuc–1a were present, but not in any other combinations, suggesting the presence of 1a–1a interactions in vivo (Figure 3C). Furthermore, we also utilized the bimolecular fluorescence complementation (BiFC) assay to verify the self-intermolecular interactions of 1a. 1a was fused to the N- and C- terminal of yellow fluorescent protein (YFP) to construct 1a–nYFP and 1a–cYFP. The two constructs were then co-infiltrated into *N. benthamiana* leaves, and the signals were observed through a confocal microscope 2 DPI. Strong yellow signals were observed in cells when both 1a–nYFP and 1a–cYFP were present, but not in other empty vector controls (Figure 3D), indicating the self-physical interaction of 1a–1a in vivo.

### 3.4. The N-Terminal of 1a Plays a Key Role in Its Inter- and Intramolecular Interactions

**Figure 4:**
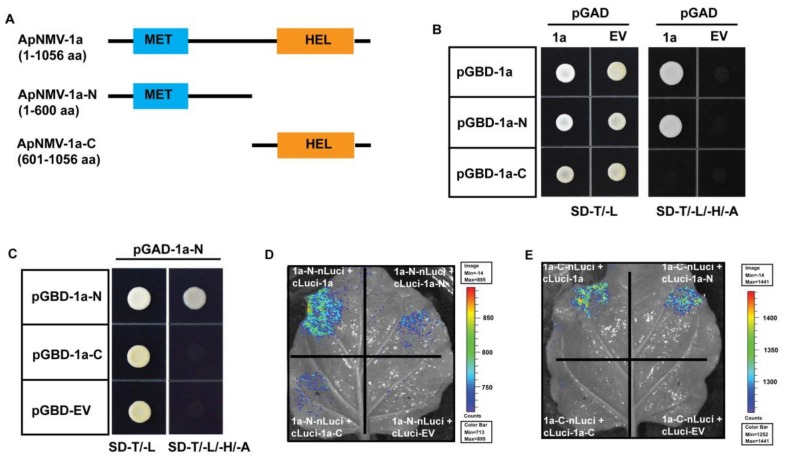
The N-terminal of 1a protein plays a key role in 1a’s intramolecular interactions. (**A**) The schematic model of split 1a protein based on the methyltransferase (MET) and helicase (HEL) domains. A yeast-two-hybrid assay suggested the existence of interactions between 1a and its N-terminal (**B**), and N-terminal with itself (**C**). (**D**) A luciferase complementation imaging assay indicated the existence of interactions of the 1a-N terminal with itself and also the 1a C-terminal. (**E**) The luciferase complementation imaging also assay suggested the interaction of the 1a C-terminal with full-length 1a and the 1a N-terminal. Empty vector (EV) served as a negative control. The bar on the right side of (D) and (E) indicated the signal intensity captured by the imaging system.

To identify the fragment that plays a key role in 1a–1a intermolecular interactions, we split the 1a protein into two portions—the N-terminal MET domain (1–600 aa) and the C-terminal HEL domain (601-1056 aa) (Figure 4A)—and constructed them into the pGAD and pGBD vectors, respectively. In the Y2H assay, we first used full-length 1a as prey and found that it only interacted with its N-terminal but not the C-terminal (Figure 4B), suggesting that the N-terminal was most likely responsible for ApNMV 1a’s intermolecular interactions. Next, we verified the interactions among the split 1a–N and –C terminal using both the Y2H and luciferase complementation imaging assay. When the 1a–N moiety was used as prey in the Y2H assay, it only interacted with itself but not with the 1a–C fragment (Figure 4C). In the luciferase assay, both interactions between 1a and 1a–N, as well as 1a–N’s self-interaction was confirmed; however, 1a–N interacted with 1a–C and could not be detected in the Y2H assay (Figure 4C,D). Specifically, when 1a–C was used as prey in the Y2H assay, it interacted with nothing (Figure S3). However, in the luciferase assay, 1a–C was found to interact with full-length 1a as well as 1a–N (Figure 4E), which were both failed to be detected in the Y2H assay. Based on data from both the Y2H and luciferase complementation imaging assay, the N-terminal of 1a interacted with itself, as well as with the C-terminal, suggesting that 1a’s N-terminal also played a key role in 1a’s intramolecular interactions.

### 3.5. ApNMV 1a Interacts with 2a^pol^

**Figure 5:**
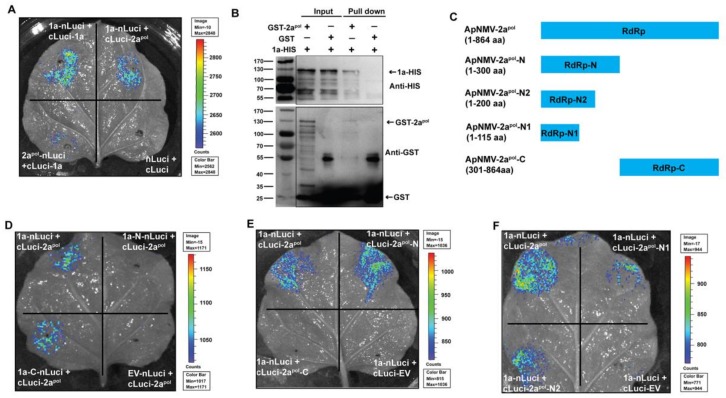
ApNMV 1a interacts with 2a^pol^. (**A**) A luciferase complementation imaging assay suggests the existence of interactions between 1a and 2a^pol^. (**B**) A pull-down assay demonstrates the presence of an interaction between 1a and 2a^pol^ in vitro. GST served as the control. Molecular mass is labeled (KDa) on the left side. GST-1a, GST, and 1a-HIS bands are indicated by arrows on the right side. (**C**) Schematic model of split 2a^pol^ that was used in the following luciferase assay. Results of luciferase complementation imaging assay in (**D**–**F**) identified the fragments that were responsible for the 1a–2a^pol^ interactions. The bar on the right side indicates the intensity of signals captured.

As mentioned previously, 1a–2a^pol^ interactions play a critical role in viral genome replication in BMV [25]. Analysis of the viral replication protein secondary structure of ApNMV and BMV revealed that the structure of ApNMV 1a was pretty similar to that of BMV 1a (Figure S4). Thus, we hypothesized that ApNMV 1a could interact with its polymerase. To verify this hypothesis, we first utilized the Y2H assay to detect the 1a–2a^pol^ interactions; viral MP and CP were also included in the assay. 1a only interacted with itself but not with any other viral components, including 2a^pol^ (Figure S5). Given the importance of 1a–2a^pol^ interactions in viral replication, we next performed a luciferase complementation imaging assay to test this interaction. We assembled 2a^pol^ to the C- and N-terminal of firefly luciferase to obtain cLuc–2a^pol^ and 2a^pol^–nLuc constructs, 1a–1a interactions served as the positive control, while empty vectors served as the negative control. We observed strong bioluminescence signals for the combination of 1a–nLuc and cLuc–1a, suggesting that 1a interacts with itself. Strong signals were also observed for the combinations of either 1a–nLuc and cLuc–2a^pol^ or cLuc–1a and 2a^pol^–nLuc; signals corresponding to the empty vectors were absent, indicating that there was an interaction of ApNMV 1a and 2a^pol^ in vivo (Figure 5A). We next utilized the pull-down assay to further verify the interaction of ApNMV 1a with 2a^pol^ in vitro. We fused a GST tag to 2a^pol^, and mixed GST–2a^pol^ with 1a–HIS and used empty GST as a negative control. The mixed proteins were eluted with a glutathione column and immunoblotted with anti-GST and anti-HIS antibodies. The results showed that 1a–HIS was pulled down in the presence of GST–2a^pol^ but not in the GST control, suggesting the presence of protein interactions of 1a–2a^pol^ in vitro (Figure 5B). Thus, we concluded that ApNMV 1a could physically interact with 2a^pol^.

To determine the fragment that was responsible for 1a–2a^pol^ interactions, we next used the luciferase complementation imaging assay to perform an experiment, as the Y2H assay was not suitable for detecting this interaction. We first inserted the split 1a and 2a^pol^ (as shown in Figure 4A and Figure 5C) into the nLuci and cLuci vectors and transformed them into the agrobacterium, followed by co-infiltration of different combinations into *N. benthamiana* leaves. When 1a–N and –C were fused to nLuci and co-infiltrated with cLuci-2a^pol^, bioluminescence signals were only observed for 1a–C but not 1a–N, suggesting that the C-terminal of 1a was responsible for interacting with 2a^pol^ (Figure 5D) The full-length 1a and empty nLuci served as the positive and negative controls, respectively. In contrast, when the N- and C-terminals of 2a^pol^ were fused to cLuci and co-expressed with 1a–nLuci, signals were only displayed for the combinations of 1a–nLuci and cLuci–2a^pol^–N, suggesting that the N-terminal of 2a^pol^ was responsible for its interaction with 1a (Figure 5E). To identify the precise fragment of 2a^pol^ that is responsible for interacting with 1a, we cut the N-terminal of ApNMV 2a^pol^ into shorter fragments (2a^pol^–N1 and –N2) and found that the first 115 aa were sufficient for maintaining its ability to interact with ApNMV 1a (Figure 5F). These data suggested that the C-terminal of 1a and the N-terminal of 2a^pol^ were responsible for the 1a–2a^pol^ interaction and that the first 115 aa of 2a^pol^ was sufficient for supporting this interaction.

## 4. Discussion

Apple mosaic disease, one of the major diseases affecting apple cultivation, severely affects apple fruit production and quality [26,27]. Two lines of evidence suggest that this disease in China is primarily caused by ApNMV, a newly identified member of *Ilarvirus* [13,14]. First, ApNMV has been detected in most apple leaves with mosaic symptoms from China [11,13,14]. Second, the mosaic symptoms in different branches of a single apple tree are correlated with the presence of ApNMV [13]. Thus, ApNMV appears to be closely associated with apple mosaic disease, at least in China, one of the major countries for apple cultivation and production in the world.

To understand the pathogenicity of ApNMV, characterizing viral protein functions is of paramount importance, especially protein interactions, either among different viral components or between viral proteins and host factors. In this study, we used ApNMV isolated from apple leaves with mosaic symptoms collected from Laiwu, Shandong Province, China, to characterize the viral protein intermolecular interactions of 1a with itself and also 2a^pol^ using multiple methods (Figure 3 and Figure 5). We further found that the N-terminal of 1a plays a key role in its inter- and intramolecular interactions (Figure 4). The C-terminal of 1a and N-terminal of 2a^pol^ are required for 1a–2a^pol^ interactions (Figure 5).

### 4.1. The ApNMV–Lw Isolate was Placed in Subgroup 3 of Ilarvirus

Similar to a previous report of the genomic structure of ApNMV [13], the genome of the ApNMV–Lw isolate consisted of three positive-strand RNA segments: RNA1 (3378 nt), RNA2 (2776 nt), and RNA3 (1910 nt). The three RNA segments encoded four viral proteins: MET and HEL domain-containing protein (1a, encoded by RNA1), an RdRp domain-containing protein (2a^pol^, encoded by RNA2), MP (encoded by the N-terminal ORF in RNA3), and CP (encoded by subgenomic RNA4) (Figure 1A). This organizational pattern of the genome is characteristic of many ilarviruses [9,28,29,30]. We also found that the ApNMV-Lw isolate had a high identity (more than 90%) to other reported ApNMV isolates (ApNMV–Qu, ApNMV–Hua, ApNMV–Hai, ApNMV–JP) at both the nucleotide and amino acid levels (Table 1). Interestingly, the identity of ApNMV–Lw with PNRSV was much higher than that of ApMV at both the nucleotide and amino acid levels (Table 1), reflecting the distinctness of ApNMV from ApMV.

The phylogenetic analysis based on nucleotides categorized the ApNMV–Lw isolate into Subgroup 3, along with other reported ApNMV isolates, in addition to ApMV and PNRSV (Figure 1B–D). Furthermore, ApNMV was closely related to ApNMV–Qu (MG924896, MG924899, and MG924902) isolated from apple trees in Zaozhuang, China (Figure 1B–D). Although all were placed in Subgroup 3, both PNRSV and ApMV were located on different branches than the ApNMV group (Figure 1B–D), confirming that ApNMV and ApMV are two different viruses despite the similarity in the symptoms that they produce in apple trees.

### 4.2. Interaction between Replication Proteins is a Conserved Feature among Tripartite RNA Viruses in Bromoviridae

Protein interactions play a key role in viral life cycles, including genome replication and virus–host interactions, to facilitate viral infection and survival in host cells. We found that the ApNMV protein 1a interacted with itself and with an RdRp domain-containing 2a^pol^ (Figure 3 and Figure 5). Furthermore, we found that the N-terminal portion plays an important role in 1a’s self-interaction, and that 1a’s C-terminal interacts with the N-terminal of 2a^pol^ (Figure 4 and Figure 5).

Similar patterns of protein interactions relating to viral replication have already been reported in BMV, which is in the genus *Bromovirus* and the family *Bromoviridae* [31]. BMV 1a, a multifunctional protein that is involved in viral genome replication and also VRC assembly, interacts with itself and also BMV 2a^pol^ [31], which is very similar to the patterns of protein interactions that we observed for ApNMV (Figure 3 and Figure 5). Specifically, the first 515 aa residues in the N-terminal of BMV 1a are required and sufficient for permitting 1a–1a interactions [31], which is consistent with our finding that the N-terminal MET domain (1–600 aa) is responsible for ApNMV 1a’s intermolecular interactions (Figure 4A,B). In addition, BMV 1a’s capping moiety also interacts with the helicase-like portion [31], and the latter even forms trimmers and larger complexes (e.g., hexamers) through intermolecular interactions [32,33]. Interactions between ApNMV 1a’s N-terminal MET domain and C-terminal HEL portion was also confirmed (Figure 4D,E), but the HEL’s self-interactions failed to be determined in our hands (Figure 4E and Figure S3). ApNMV 1a’s C-terminal was not observed to interact with itself nor with 1a’s N-terminal in the Y2H assay. This observation likely stems from the fact that the C-terminal protein is not successfully expressed in yeast cells. Thus, additional work, such as immunoblotting with 1a’s specific antibody, is needed to verify this finding. Lastly, 1a–1a interactions have also been reported in the cucumber mosaic virus (CMV) and cowpea chlorotic mottle virus (CCMV) [31,33], both tripartite RNA viruses in *Cucumovirus* and *Bromovirus*, respectively. Given the 1a–1a interactions that we verified in ApNMV and those already documented in BMV, CMV, and CCMV, 1a’s self-interaction may be a conserved feature of tripartite viruses in the *Bromoviridae*.

In addition to 1a self-interactions, BMV 1a also interacts with 2a^pol^. Specifically, the N-terminal 115 aa of 2a^pol^ and the C-terminal HEL-like domain are necessary and sufficient for BMV 1a–2a^pol^ interactions [34,35], which is consistent with our finding in ApNMV that the C-terminal of 1a and the N-terminal of 2a^pol^ (1-115 aa) are required for 1a–2a^pol^ interactions (Figure 5F). However, 1a–2a^pol^ interactions were not detected in Y2H assay in our hands, likely because the 2a^pol^ protein could not be successfully expressed in yeast cells. In addition, ApNMV 1a and 2a^pol^ were observed to be co-localized in the cytoplasm of plant cells under a confocal microscope (Figure 2B), which is consistent with the cytoplasm colocalization of BMV 1a and 2a^pol^ in protoplasts of barley, the natural host of BMV [36,37]. Furthermore, immunostaining in protoplasts of natural host and yeast cells revealed that the two replication proteins of BMV co-localized on endoplasmic reticulum (ER), where viral RNA synthesis takes place [36,37,38]. Given that the replication of all well-studied positive-strand RNA viruses is associated with intracellular membranes, we utilized KDEL–RFP as an ER marker and found ApNMV 1a and 2a^pol^ only partially co-localized with ER membranes (Figure 2D). The different distribution patterns of BMV and ApNMV proteins may be caused by the different detection methods. BMV 1a and 2a^pol^ specific antibodies were utilized to detect viral protein localization in natural host cells [38], while eGFP fused ApNMV 1a and 2a^pol^ were utilized to detect their distribution in *N. benthamiana* (Figure 2A,D). The fused GFP protein probably affected the conformation of viral proteins, and led to unprecise localization. Thus, specific antibodies and the viral natural host protoplasts are needed to determine the precise location of ApNMV 1a, 2a^pol^, as well as the viral replication sites. In sum, the interaction between replication proteins, including the inter- and intramolecular interactions of 1a and 1a–2a^pol^, is a conserved feature among tripartite RNA viruses in the *Bromoviridae*.

### 4.3. Interactions between Replication Proteins are Critical for Genome Replication and VRC Assembly of Tripartite RNA Viruses

Given their relatively small genomes, viruses have evolved to maximize the function of their viral proteins. BMV has become a model for studying genome structure, virus–host interactions, and protein functions of positive-strand RNA viruses as it is the type member of *Bromoviridae* and also the representative member of the alphavirus-like superfamily that includes viruses infecting humans, animals, and plants [23]. In the BMV life cycle, viral protein 1a plays a key role in multiple processes, especially in the formation of the VRC and genome replication. Generally, 1a first interacts with 2a^pol^ in the cytoplasm. 1a contains an N-terminal Helix A motif, which is a small amphipathic α-helix that is crucial for 1a’s membrane association, 1a-induced membrane rearrangements, as well as 1a-mediated recruitment of viral RNA templates and 2a^pol^ [25]. Through the N-terminal Helix A motif, 1a targets the outer ER membrane to induce the formation of spherules with the aid of multiple host factors [25,39]. 1a then recruits RNA templates into spherules and catalyzes the production of negative-strand RNAs along with 2a^pol^ [25,40,41]. The interactions of 1a–1a and 1a–2a^pol^ are critical for these processes. For instance, the formation of spherules that support successful RNA replication relies on the direct linkage and coherent action of 1a’s self-interacting capping and helicase portion [32]. Because neither the capping domain nor the helicase domain can induce normal spherules to support viral RNA replication, the co-expressed capping domain displays dominant-negative effects that disturb the induction of replication vesicles induced by full-length 1a in yeast cells [32]. A three-amino-acid insertion in the BMV 1a protein sequence inhibited BMV RNA replication in vivo [42]. In addition, this insertion also blocked its interaction with 2a^pol^ in vitro, which, in turn, suggests that 1a–2a^pol^ interactions play a critical role in BMV genome replication [43]. Thus, given the similar genomic structure and protein secondary structure to BMV (Figures S2 and S4), ApNMV 1a may also play a key role in multiple processes of the ApNMV life cycle, such as the genome replication and VRC assembly; however, additional work is needed to verify this hypothesis.

In sum, using an ApNMV–Lw isolate obtained from Laiwu of China, we found that ApNMV 1a interacts with itself and with the viral RdRp domain-containing protein 2a^pol^. The N-terminal MET portion of la is required for its self-interaction, and its C-terminal HEL domain interacts with the N-terminal of 2a^pol^. Our findings provide insight into the interactions among viral proteins of ApNMV, which is a novel ilarvirus associated with apple mosaic disease in China. In addition, the results of our study shed light on the mechanism underlying the pathogenicity of ApNMV.

## Figures and Tables

**Table 1:** Sequence similarity of ApNMV–Lw with other ApNMV isolates, apple mosaic virus (ApMV), and prunus necrotic ringspot virus (PNRSV).

		ApNMV-Qu	ApNMV-Hua	ApNMV-Hai	ApNMV-JP	ApMV	PNRSV
nt %	RNA1	97.37	94.00	97.17	96.33	59.44	64.00
	RNA2	96.45	94.91	93.55	94.23	58.02	58.20
	RNA3	96.10	88.47	93.04	91.57	49.73	59.39
aa %	1a	99.53	99.05	99.24	98.67	63.72	69.60
	2a^pol^	96.76	94.56	96.06	93.29	57.08	60.07
	MP	96.79	92.86	94.29	93.21	50.70	55.83
	CP	96.35	94.52	95.89	95.89	40.54	56.83

Note that the viruses used in this table are the same as those used in Figure 1B–D.

## Supplementary Materials

The following are available online at https://www.mdpi.com/1999-4915/12/4/474/s1.
