# Supplementary material for: Determination of Protein Interactions among Replication Components of Apple Necrotic Mosaic Virus
Source: Viruses. 2020 Apr 22;12(4):474. doi: 10.3390/v12040474 (PMC7232516; doi:10.3390/v12040474)
Supplement: Supplementary file 1 [file viruses-12-00474-s001.zip › Supplemental Figure Legend.docx]

**Supplemental Fig. S1 2a^pol^-RFP localized to cytoplasm in epidermal cells of *N. benthamiana* leaves.** Upper panel displayed the distribution of empty red fluorescent protein (RFP) in both cytoplasm and nucleus in epidermal cells of *N. benthamiana* leaves. BF, bright field; Scale bar, 10 μm.

**Supplemental Fig. S2 Amino acids sequence analysis of ApNMV-1a (A) and BMV-1a (B) to show the structural similarity of the two proteins.** The schematic models were obtained by using the BLAST tool on the website of National Center for Biotechnology Information.

**Supplemental Fig. S3 Using yeast-two-hybrid assay to detect the interaction of 1a-C with splitted 1a fragments.** Splitted ApNMV-1a (1a-N and 1a-C) was fused to either the activation domain (pGAD) or the DNA binding domain (pGBD) of GAL4 system to test their interactions. Empty pGBD vector served as control.

**Supplemental Fig. S4 Secondary structure analysis of 1a, 2a^pol^ of both ApNMV and BMV.** Tables on the left side showed the percentage of each kind of secondary structure.

**Supplemental Fig. S5 Using yeast-two-hybrid assay to detect the interaction of 1a with other viral components.** ApNMV-1a was fused to either the activation domain (pGAD-1a) or the DNA binding domain (pGBD-1a) of GAL4 system to test its interaction with other viral components. Empty pGAD and pGBD vectors served as control.
