# Supplementary material for: Determination of Protein Interactions among Replication Components of Apple Necrotic Mosaic Virus
Source: Viruses. 2020 Apr 22;12(4):474. doi: 10.3390/v12040474 (PMC7232516; doi:10.3390/v12040474)
Supplement: Supplementary file 1 [file viruses-12-00474-s001.zip › Supplementary Table S1.docx]

**Supplemental Table S1 Primer for plasmids construction.**

Y2H: yeast two-hybrid, PD: pull-down, BiFC: bimolecular fluorescence complementation,

Luci: luciferase complementation imaging

F: Forward primer, R: reverse primer

| Gene | Primers |
| --- | --- |
| 1a-Y2H | F: 5’ GgaattcATGGACCCAGTTGCTTTATCCGATTTGC 3’ |
| 1a-Y2H | R: 5’ AActgcagCAATTTGTAATCGGTATGTAAGTAGCTAATGACTCGC 3’ |
| 1a-N-Y2H | R: 5’ AActgcagGACAACCTGTTCTCGGATATCTACCAGG 3’ |
| 1a-C-Y2H | F: 5’ GgaattcGAGAAACCAAGTGATTTGGTTGTGAATAAGTACGC 3’ |
| 2a-Y2H | F: 5’ TCCcccgggGATGGACCCACTCTACCATCTCGTG 3’ |
| 2a-Y2H | R: 5’ CGggatccTCGGGGTTCGGTCGTGTTCATCTC 3’ |
| 1a-eGFP | F: 5’ CGggatccATGGACCCAGTTGCTTTATCCGATTTGC 3’ |
| 1a-eGFP | R: 5’ GCtctagaCAATTTGTAATCGGTATGTAAGTAGCTAATGACTCGC 3’ |
| 2a-eGFP | F: 5’ CGggatccATGGACCCACTCTACCATCTCGTG 3’ |
| 2a-eGFP | R: 5’ GCtctagaTCGGGGTTCGGTCGTGTTCATC 3’ |
| CP-Y2H | F: 5’ GgaattcATGGTGTGCAACCGCTGTCATC 3’ |
| CP-Y2H | R: 5’ AActgcagCTAGACATCCAAAAGGTCTTCATCGACC 3’ |
| MP-Y2H | F: 5’ GgaattcATGGCAGTAGCTGACTTGAAATCTTCTTCAG 3’ |
| MP-Y2H | R: 5’ AActgcagTCAAGCATTTATAGGATCGGCCCTCACG 3’ |
| 1a-BiFC | F: 5’ CGggattcATGGACCCAGTTGCTTTATCCGATTTGC 3’ |
| 1a-BiFC | R: 5’ GGggtaccCAATTTGTAATCGGTATGTAAGTAGCTAATGACTCGC 3’ |
| 1a-Luci | F: 5’ GCtctagaATGGACCCAGTTGCTTTATCCGATTTGC 3’ |
| 1a-Luci | R: 5’ CCGctcgagCAATTTGTAATCGGTATGTAAGTAGCTAATGACTCG 3’ |
| 2a-Luci | F: 5’ GCtctagaATGGACCCACTCTACCATCTCGTG 3’ |
| 2a-Luci | R: 5’ CCGctcgagTCGGGGTTCGGTCGTGTTCATC 3’ |
| 1a-C-Luci | F: 5’ GCtctagaGAGAAACCAAGTGATTTGGTTGTGAATAAGTACGC 3’ |
| 1a-N-Luci | R: 5’ CCGctcgagGACAACCTGTTCTCGGATATCTACCAGG 3’ |
| 2a-N1-Luci | R: 5’ CCGctcgagCTGTTCGTACATACTGACTGACTGTCGAG 3’ |
| 2a-N2-Luci | R: 5’ CCGctcgagCATGTCTGTTTCTACTTCGATTGTTAGATCG 3’ |
| 2a-N3-Luci | R: 5’ CCGctcgagATCTATAGCCATCTGGATGAATTCGGG 3’ |
| 2a-C-Luci | F: 5’ GCtctagaGAATTGTTACCAGGTGTTGCTGATGTCG 3’ |
| 1a-PD | F: 5’ CGgaattcATGGACCCAGTTGCTTTATCCGATTTGC 3’ |
| 1a-PD | R: 5’ CCGctcgagCAATTTGTAATCGGTATGTAAGTAGCTAATGACTCG 3’ |
| 2a-PD | F: 5’ CGggatccATGGACCCACTCTACCATCTCGTG 3’ |
| 2a-PD | R: 5’ CCGctcgagTCGGGGTTCGGTCGTGTTCATC 3’ |
