# Supplementary figures and images for: Determination of Protein Interactions among Replication Components of Apple Necrotic Mosaic Virus
Source: Viruses. 2020 Apr 22;12(4):474. doi: 10.3390/v12040474 (PMC7232516; doi:10.3390/v12040474)

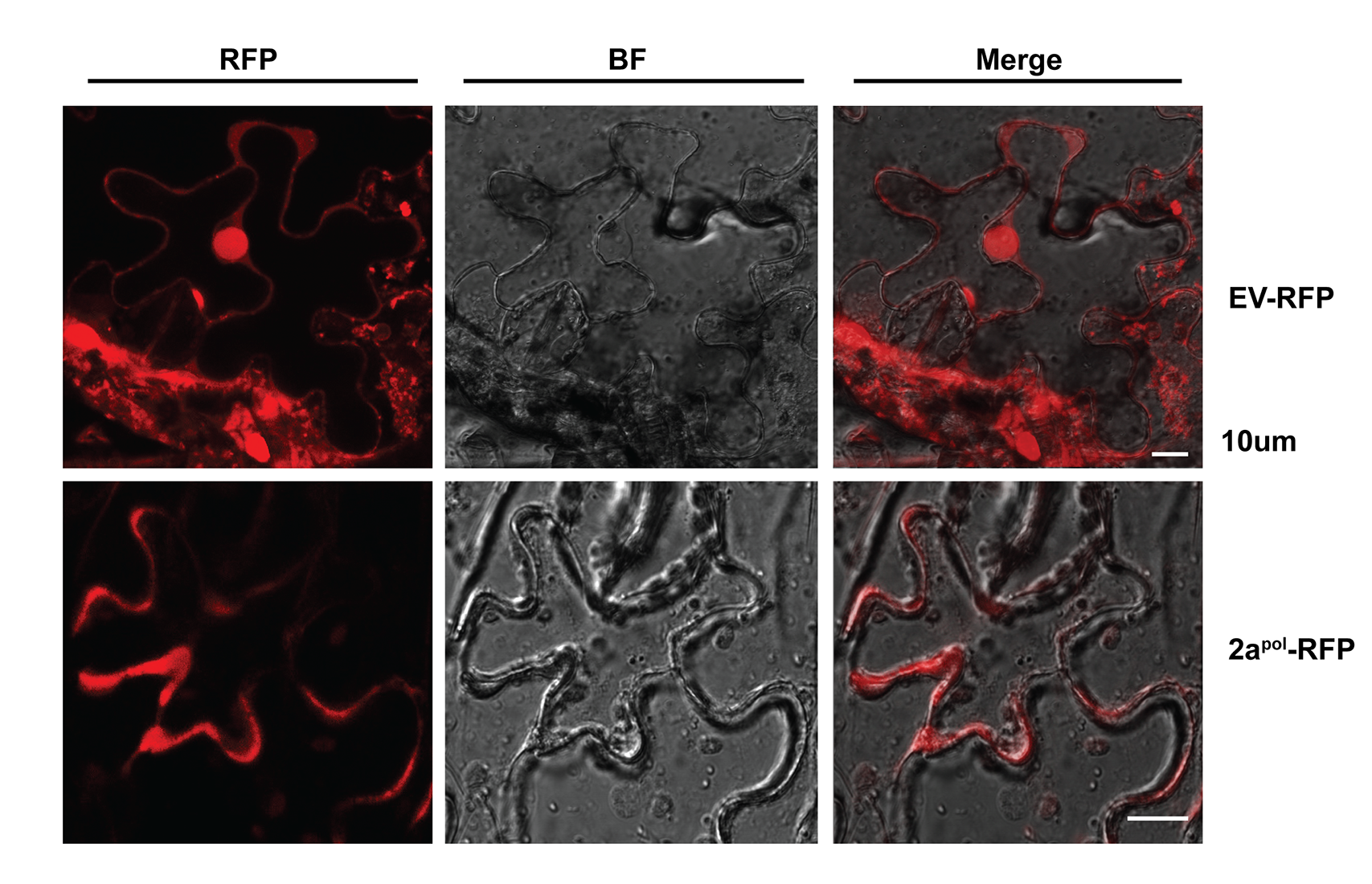

Supplement: Supplementary file 1 [file viruses-12-00474-s001.zip › Supplemental Fig S1.tif]

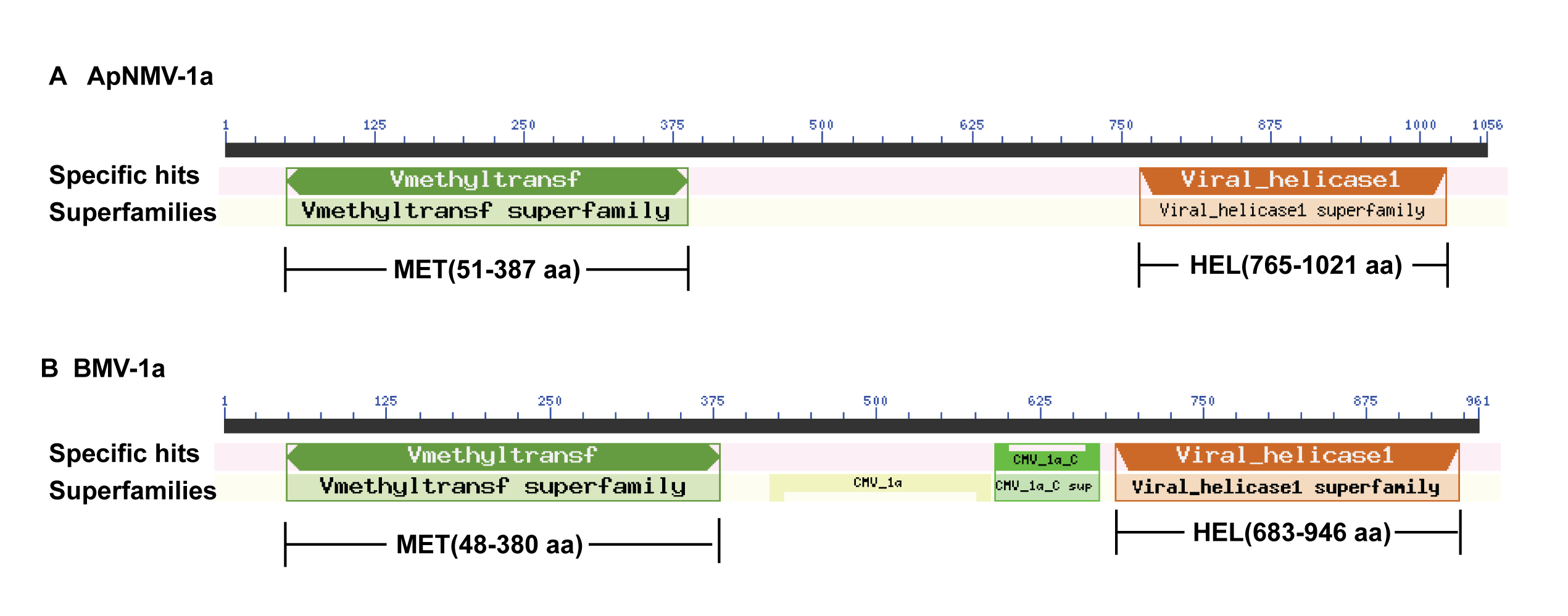

Supplement: Supplementary file 1 [file viruses-12-00474-s001.zip › Supplemental Fig S2.tif]

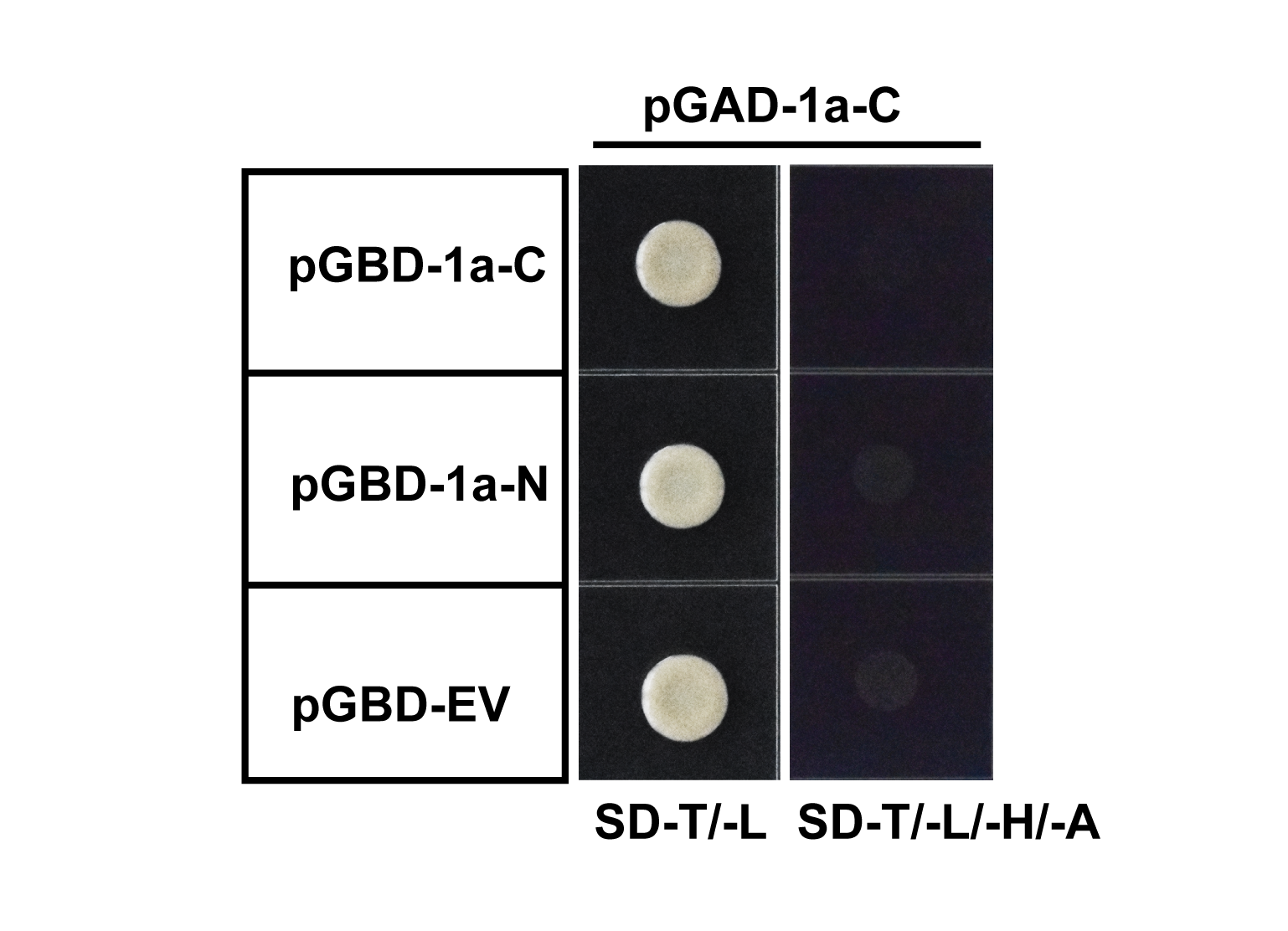

Supplement: Supplementary file 1 [file viruses-12-00474-s001.zip › Supplemental Fig S3.tif]

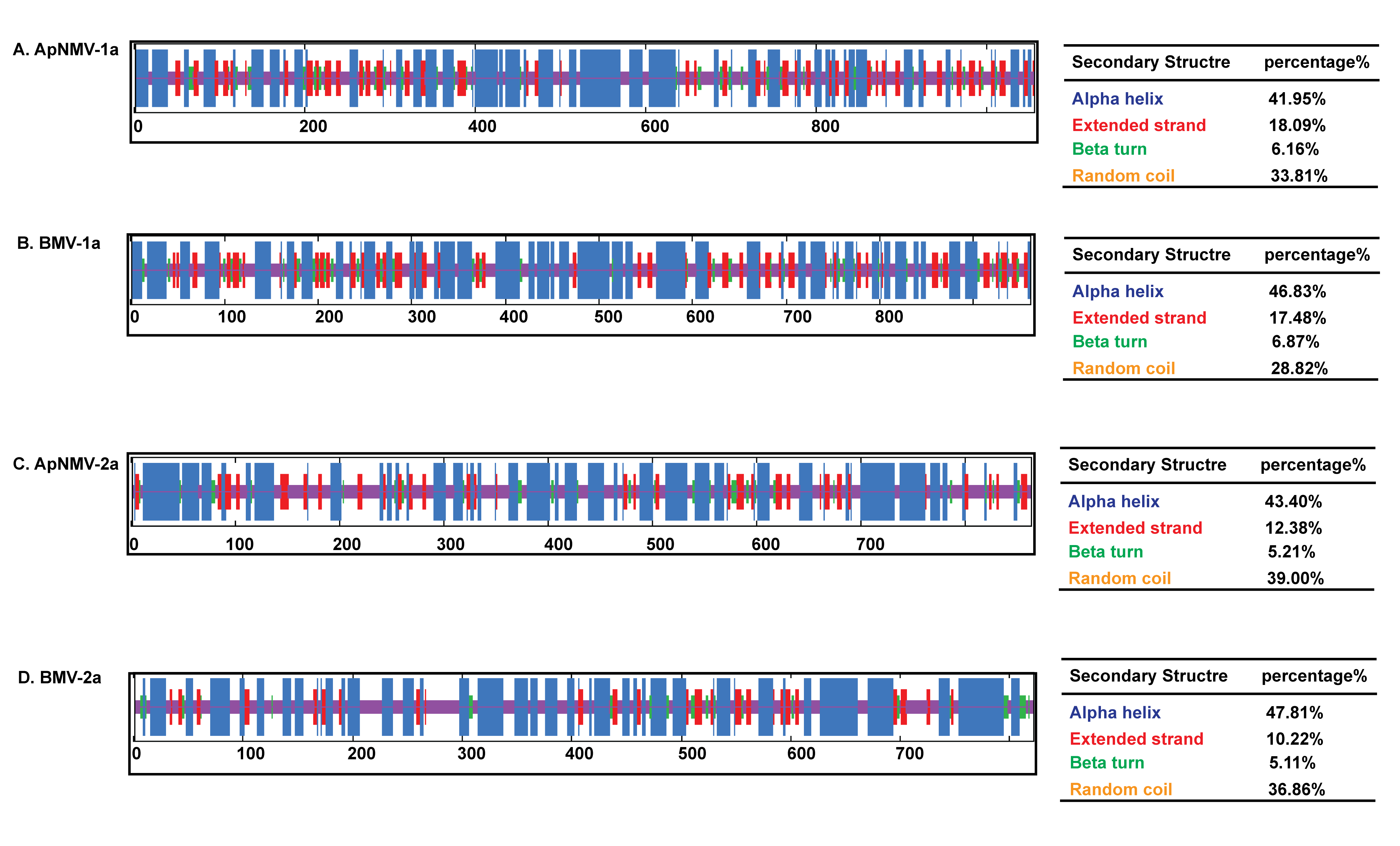

Supplement: Supplementary file 1 [file viruses-12-00474-s001.zip › Supplemental Fig S4.tif]

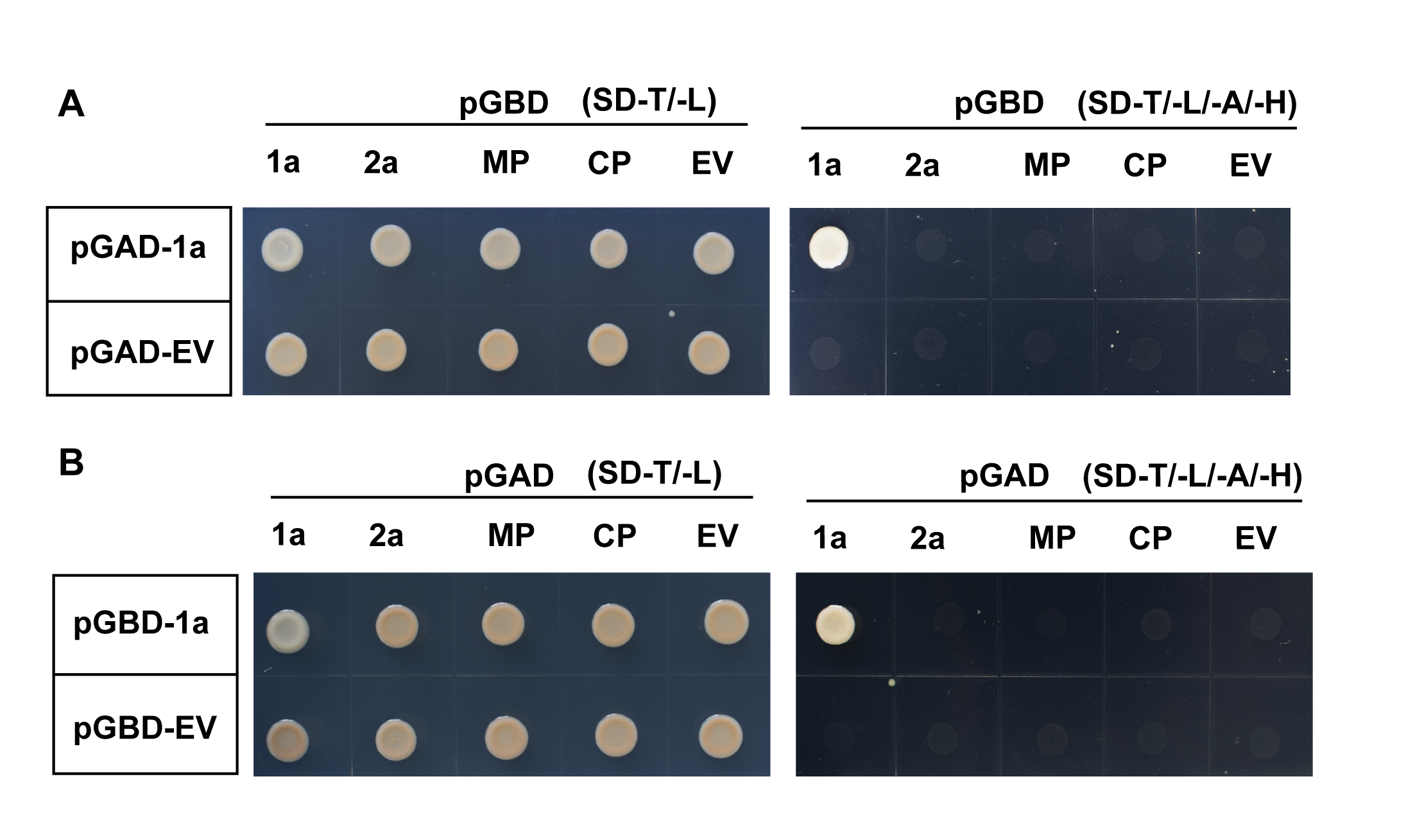

Supplement: Supplementary file 1 [file viruses-12-00474-s001.zip › Supplemental Fig S5.tif]
