# Supplementary material for: Determination of Protein Interactions among Replication Components of Apple Necrotic Mosaic Virus
Source: Viruses. 2020 Apr 22;12(4):474. doi: 10.3390/v12040474 (PMC7232516; doi:10.3390/v12040474)
Supplement: Supplementary file 1 [file viruses-12-00474-s001.zip › Supplementary Table S2.doc]

**Supplemental Table S2 Primers for ApNMV cloning**

F: Forward primer, R: reverse primer

| Gene | Primers |
| --- | --- |
| RNA1 | F: 5’ ATGGACCCAGTTGCTTTATCCGAG 3’ |
| RNA1 | R: 5’ CTACAATTTGTAATCGGTATGTAAGTAGCTAATGACTCG 3’ |
| RNA2 | F: 5’ ATGGATCCACTCTACCATCTCGTGAAGC 3’ |
| RNA2 | R: 5’ CTAACCAGTTTTCTTCGCAATCGACC 3’ |
| RNA3 | F: 5’ ATGGCAGTAGCTGACTTAAAAACCTCTTC 3’ |
| RNA3 | R: 5’ CTAGACATCCAAAAGGTCTTCATCGACC 3’ |
| RNA1-GSP1 | R: 5’ GCCATCACGTACTCGGCCTTCCTGTCAC 3’ |
| RNA1-GSP2 | F: 5’ GTGTGTACGCCGCGGCCACTTTGGCG 3’ |
| RNA2-GSP1 | R: 5’ CGAGGGTCGATTCATCCGGACTCTCGG 3’ |
| RNA2-GSP2 | F: 5’ GGATTTAGCACCCGAGGCTTTCGATGCTTGG 3’ |
| RNA3-GSP1 | R: 5’ CATCGGGACATATGTCCGTCCCTTCGGCC 3’ |
| RNA3-GSP2 | F: 5’ GGTCCGAATGAGGTACCACGAGTACCGAGG 3’ |
| Long Primer | 5’ TAATACGACTCACTATAGGGCAAGCAGTGGTATCAACGCAGAG 3’ |
| Short Primer | 5’ CTAATACGACTCACTATAGGGC 3’ |
